# Affective attention under cognitive load: reduced emotional biases but emergent anxiety-related costs to inhibitory control

**DOI:** 10.3389/fnhum.2013.00188

**Published:** 2013-05-13

**Authors:** Nick Berggren, Anne Richards, Joseph Taylor, Nazanin Derakshan

**Affiliations:** ^1^Department of Psychological Sciences, Birkbeck University of LondonLondon, UK; ^2^St Johns College Research Centre, St Johns College, University of OxfordOxford, UK

**Keywords:** cognitive load, trait anxiety, threat processing, visual attention, antisaccade task

## Abstract

Trait anxiety is associated with deficits in attentional control, particularly in the ability to inhibit prepotent responses. Here, we investigated this effect while varying the level of cognitive load in a modified antisaccade task that employed emotional facial expressions (neutral, happy, and angry) as targets. Load was manipulated using a secondary auditory task requiring recognition of tones (low load), or recognition of specific tone pitch (high load). Results showed that load increased antisaccade latencies on trials where gaze toward face stimuli should be inhibited. This effect was exacerbated for high anxious individuals. Emotional expression also modulated task performance on antisaccade trials for both high and low anxious participants under low cognitive load, but did not influence performance under high load. Collectively, results (1) suggest that individuals reporting high levels of anxiety are particularly vulnerable to the effects of cognitive load on inhibition, and (2) support recent evidence that loading cognitive processes can reduce emotional influences on attention and cognition.

## Introduction

Efficient goal-directed behavior depends upon top-down attentional control, allowing goal-relevant information to be attended to rather than irrelevant information. However, the efficiency with which we employ attentional control depends upon a combination of both internal factors, one's inherent attentional control abilities, and external factors, the amount a task or goal taxes our attentional control abilities in order to accomplish. One internal factor that has been shown to affect attentional control is an individual's self-reported level of trait anxiety (Eysenck and Derakshan, 2011).

It has been well-documented that trait anxious individuals show a bias in their selective attention toward irrelevant threat-laden information, with a meta-analysis finding consistent evidence of such a bias (Bar-Haim et al., 2007). For example, dot probe paradigms demonstrate that highly anxious individuals show markedly increased latency costs when ignoring threatening stimuli and responding to a target at a different spatial location (e.g., Arndt and Fujiwara, 2012). Such biases could be argued to be hardwired, even extending to individual differences in amygdala response to subliminal threat items (Etkin et al., 2004).

Based on this evidence, one potential conclusion is that anxiety does not affect attentional control in general, but selectively biases attentional control in response to the presentation of threatening stimuli. A growing body of work, however, has highlighted that anxious individuals also show impaired attentional control in situations where threat is absent. For example, trait anxious individuals show greater costs on latency performance in the antisaccade task (Derakshan et al., 2009; Ansari and Derakshan, 2011b) and increased response-competition in flanker tasks (Bishop, 2009; Pacheco-Unguetti et al., 2010). These findings can be accommodated within Attentional Control Theory (ACT; Eysenck et al., 2007), which posits that trait anxiety disrupts the three key facets of attentional control: inhibition of task-irrelevant information, flexibly shifting attention, and updating representations in working memory. Accumulating behavioral, electrophysiological, and neuroimaging evidence supports these predictions in recent years (see Eysenck and Derakshan, 2011; Berggren and Derakshan, 2013, for reviews).

According to ACT the effects of anxiety on attentional control should be greater under competing task demands. Indeed, it has been well-documented that the level of external task demands can strongly influence attentional control. This has most commonly been manipulated through varying cognitive demands on working memory during study via a secondary task such as item rehearsal. Such a manipulation is believed to tax executive resources, required for maintaining task goals and prioritizing task-relevant over irrelevant information (Baddeley, 1986). Loading working memory increases response latencies and error rates in the antisaccade task (Roberts et al., 1994; Kane et al., 2001; Berggren et al., 2011), and increases task-irrelevant interference by both response-competing and wholly irrelevant distractor items (de Fockert et al., 2001; Lavie et al., 2004; Lavie and De Fockert, 2005). Distractor interference also increases under load across modalities, impacting processing in the auditory and tactile domains (Dalton et al., 2009a,b). In particular, de Fockert et al. (2001) showed that loading working memory increased activity in the visual cortex, for face distractors in the fusiform “face” area, suggesting that attentional selection and control are strongly influenced by the availability of working memory resources. Finally, the ability to inhibit distractor items, measured by negative priming for distractors that subsequently become targets, is eliminated when cognitive processes are taxed by load (de Fockert et al., 2010).

A number of studies have investigated whether cognitive load may particularly hamper attentional control in anxious individuals, disrupting task performance. These studies can be roughly divided into two subsets: those investigating the effect of cognitive load on distraction in the presence of task-irrelevant emotional material, and those assessing effects on distraction in the absence of emotional stimuli. For the former, evidence has been inconsistent. Studies examining fear-potentiated startle reflex have suggested that enhanced distraction in anxiety is reduced under cognitive load (Dvorak-Bertsch et al., 2007; Vytal et al., 2012), investigations examining the late positive potential (LPP; associated with emotional arousal) have implied smaller reductions under load in anxious individuals (MacNamara et al., 2011), and studies measuring distraction by emotional faces have found increased vigilance in anxiety under load (Ladouceur et al., 2009; Judah et al., 2013). However, these effects may be confounded by the influence of cognitive load on emotion processing in general. While non-emotional distraction appears to increase under cognitive load, as outlined above, emotional distraction appears to generally be reduced as indexed by emotional startle (King and Schaefer, 2011), LPP (MacNamara et al., 2011; Van Dillen and Derks, 2012) and RT distraction (Van Dillen and Koole, 2009). Thus, reductions in anxious threat biases may reflect a more general impact of cognitive load on emotion processing.

Further insight into how cognitive load affects attentional control in anxiety was obtained by Berggren et al. (2012) who employed a visual search paradigm where participants responded to a target face of a different emotional expression to a crowd (e.g., a neutral face among a crowd of happy faces). Cognitive load was induced by participants simultaneously counting back in threes from a specified number at the start of each trial. Low and high anxious participants did not differ in their performance under no-load, but while low anxious participants showed no performance cost with the introduction of counting, the high anxious participants were significantly slower. This suggested that cognitive load had a more potent effect on individuals with high anxiety, and notably this effect occurred regardless of the emotional content of distractor faces. However, this visual search paradigm contained no direct form of distraction; displays contained a target with a number of non-target items, but these additional stimuli could not be directly examined for the extent to which they impeded task performance. Thus, one could argue that group differences under cognitive load may simply be due to a general slowing of reaction time not indicative of hampered inhibitory control *per se*. In other words, anxious individuals may simply have demonstrated performance costs due to task demands rather than any effect on attentional control aspects of inhibition.

In the present study, we aimed to build upon previous work using a task containing task-irrelevant information and requiring cognitive inhibition, thus enabling a clearer test for the prediction that cognitive load should disrupt attentional control to a greater extent in high anxious individuals. We utilized the antisaccade task where participants are required to shift their overt attention toward or away from an abrupt visual onset, the latter process requiring cognitive inhibition to suppress a reflexive occulomotor response (Ettinger et al., 2008). Both anxiety and cognitive load have been previously shown to increase latencies on “look away” antisaccade trials (Derakshan et al., 2009; Berggren et al., 2011), while having no effect on “look at” prosaccade trials that require no inhibitory processing. Thus, the current study disentangled the possibility that load increases response latencies, as effects should be confined to antisaccade trials weighting on attentional control. We manipulated load using a previously demonstrated method (Berggren et al., 2011); participants heard three kinds of auditory tones while completing the antisaccade task, and responded simply with the word “tone” on low load trials or the words “high,” “mid,” or “low” depending on the tone's pitch in high load. We hypothesized that load should increase antisaccade eye-movement latencies, while having no effect on reflexive prosaccade latencies. In addition, based on ACT's predictions, we hypothesized that the load cost on antisaccades would be exacerbated for individuals reporting high levels of trait anxiety.

Finally, we also manipulated the emotional valence of visual onsets signaling participants to make an eye-movement saccade in the antisaccade task, using facial stimuli of different expressions (neutral, happy, and angry). We aimed to further explore whether distraction from emotional faces would be reduced under cognitive load as suggested in previous work, particularly for threatening angry/fearful stimuli (e.g., King and Schaefer, 2011). We also examined how this effect could be modulated by trait anxiety levels, in light of the wealth of literature that anxiety should enhance distraction by threat-related content as well as distraction by non-emotional information generally.

## Method

### Participants

Ninety-four participants (29 males; mean age = 29 years, *SD* = 6) were recruited via advertisements posted in University of London departments. All participants had normal hearing, normal or corrected-to-normal vision, and were naïve to the experimental hypotheses.

### Apparatus, materials, and stimuli

An SR Research Eyelink 1000 eye-tracker (SR Research, ON, Canada) was used to record eye-movements, tracking one eye. Nine-point calibration ensured that tracking accuracy was within 1° of visual angle. Stimuli were presented on a 21 inch Viewsonic CRT monitor (140 Hz), and viewing distance was held constant at 60 cm using a chinrest. The experiment was created using the SR Research Experiment Builder software. A laptop played auditory tones separately during blocks, presented in a randomized order using E-Prime software (Schneider et al., 2002). Face stimuli of neutral, angry and happy expressions were selected from the NimStim Face Stimulus Set (Tottenham et al., 2009) and the Ekman series (Ekman and Friesen, 1976). Six separate identities were used in total, with a 1:1 gender ratio. All face images were cropped and modified to only show the face and appear in black and white. For saccade recording, an amplitude of 2° each side of fixation was used as saccade boundaries; eye-movements crossing either boundary were recorded for latency and accuracy.

### Procedure

**Figure 1:**
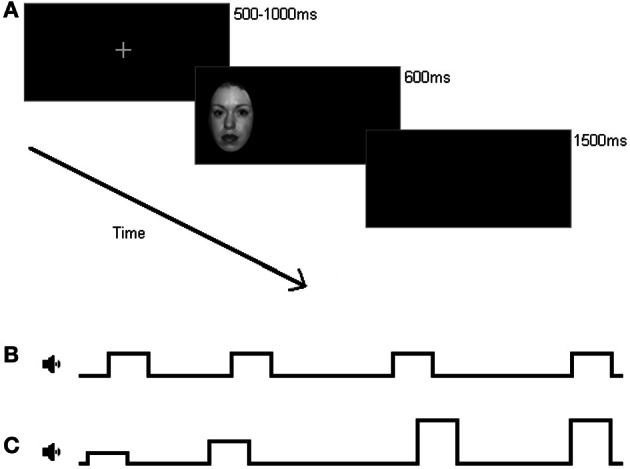
**Example trial display (not to scale). (A)** Following fixation, a face image appeared either in the left or right periphery, displaying either a neutral, angry, or happy emotional expression. Depending on the block type, participants were asked to move their eyes from fixation to the image as quickly as possible, or look away from the image and move their eyes to the opposite end of the screen. **(B)** In addition to eye movements, participants simultaneously responded verbally to tones played during blocks. Under low load, participants always heard a mid-pitched tone, and responded by saying “tone” each time they heard a sound. **(C)** Under high load, tones were presented at three different pitches, and participants responded to this with “low,” “mid,” or “high” when a tone played. All tones played randomly every 1900–2300 ms.

The study was approved by the departmental ethics committee. The experiment was conducted within a sound-protected room. After providing consent, participants completed the State-Trait Anxiety Inventory^1^ (STAI; Spielberger et al., 1983), a reliable measure of self-report trait anxiety level through the sum of scaled multiple choice responses (Spielberger et al., 1995), before being given the experimental instructions. Each trial began with a fixation cross (approximately 0.67 × 0.67° of visual angle) in the center of the screen appearing for up to 1000 ms. Participants were instructed to fixate this cross and, once they had fixated between 500 and 1000 ms after its onset, the trial moved forward immediately acting as a drift correct to tracking. Following this, face stimuli subtending approximately 3.44 × 5.25° appeared in the left or right periphery (at an eccentricity of approximately 11.23° from stimulus center to fixation) for 600 ms. At the start of each block participants were signaled how they should respond to the stimuli; in prosaccade blocks, participants were asked to move their eyes and fixate the face stimuli as quickly and as accurately as possible. In antisaccade blocks, participants were told to move their eyes away from the face stimuli to the mirror location in the other periphery as quickly and as accurately as possible. It was also emphasized that on antisaccade trials, participants should try their best to avoid looking at the face stimuli. A 1500 ms inter-trial interval was used (see Figure 1 for details).

Auditory tones played via a laptop provided the secondary load manipulation. In high load blocks, participants heard three differently-pitched tones presented every 1900–2300 ms (at any possible increments of 100 ms). Participants verbally responded to whether the pitch of the tone was “low,” “mid,” or “high” while continuing to complete the anti/prosaccade task. In low load blocks, participants only ever heard the middle pitched tone, and were simply required to respond by saying the word “tone” whenever they heard one. Participants were told to respond promptly and accurately to the tones, and the experimenter monitored their performance, giving feedback at the end of blocks if errors had been made.

There were thus four different blocked conditions, prosaccade/antisaccade with low/high cognitive load. Facial expression of visual onsets in the task was presented randomly within blocks and each expression appeared on an equal number of trials. Participants were given initial practice at discriminating the auditory tones, and subsequently 16 practice trials in the antisaccade/prosaccade task. Participants then completed eight blocks of 36 trials, with block order following an ABCDDCBA format (condition to letter counterbalanced across participants). Participants were then thanked and debriefed.

## Results

### Within-subjects effects

Data from 10 participants were removed due to overall antisaccade errors of over 50%. Anticipatory saccade (eye movements quicker than 85 ms; *M* = 1.74% of data) and no recorded saccade trials (*M* = 1.65% of data) were also removed from the data prior to error rate analysis.

#### Saccade latencies

Correct response latencies from 84 participants were entered into a three-way ANOVA with the factors expression (neutral, angry, happy), load (low, high), and condition (antisaccade, prosaccade). Descriptive statistics are shown in Table 1. Analysis showed a main effect of load [*F*_(1, 83)_ = 24.52, *p* < 0.001], indicating slower saccade latencies under high load (*M* = 251 ms, *SD* = 29) vs. low load (*M* = 243 ms, *SD* = 33). A main effect of condition [*F*_(1, 83)_ = 495.74, *p* < 0.001] also demonstrated faster latencies on prosaccade (*M* = 200 ms, *SD* = 30) compared with antisaccade trials (*M* = 294 ms, *SD* = 40). Importantly, a two-way interaction of load × condition [*F*_(1, 83)_ = 18.54, *p* < 0.001] revealed that load increased latencies on antisaccade trials [*M* = 286–302 ms; *t*_(83)_ = 6.36, *p* < 0.001], but did not affect prosaccade speed (*M* = 200–201 ms; *t* < 1).

**Table 1:** **Mean saccadic latencies (milliseconds) and percentage error rates within expression, load, and condition factors (standard deviation of the mean in parentheses)**.

**Expression**	**Level of load and condition**			
	**Low load**		**High load**	
	**Antisaccade**	**Prosaccade**	**Antisaccade**	**Prosaccade**
Neutral	286 (45)	199 (34)	302 (42)	202 (35)
	16 (14)	5 (7)	20 (14)	4 (7)
Angry	290 (49)	202 (38)	299 (46)	201 (34)
	18 (13)	5 (7)	19 (13)	6 (9)
Happy	281 (43)	200 (35)	305 (48)	200 (31)
	15 (13)	6 (8)	20 (14)	5 (8)

There was no main effect of expression, or an interaction of expression × condition (*F*'s < 1). However, there was both a significant two-way interaction of expression × load [*F*_(2, 166)_ = 5.25, *p* < 0.01], and a Three-Way interaction of expression × load × condition [*F*_(2, 166)_ = 3.68, *p* < 0.03]. To decompose these effects, separate ANOVAs were firstly conducted within each level of the condition factor. For prosaccades, there was no main effect of expression (*F*'s < 1), and no two-way interaction with load [*F*_(2, 166)_ = 1.45, *p* = 0.24]. On antisaccade trials, there was no main effect of expression (*F* < 1), but a significant interaction with load did emerge [*F*_(2, 166)_ = 5.74, *p* < 0.01]. One-Way ANOVAs showed no significant effect of expression under high load [*F*_(2, 166)_ = 1.14, *p* = 0.32], but a significant effect under low load [*F*_(2, 166)_ = 5.91, *p* < 0.01]. As reflected in Table 1, latencies were fastest in response to happy expressions and slowest for angry, corresponding to a strong linear contrast [*F*_(1, 83)_ = 11.6, *p* = 0.001]. Pairwise comparisons showed a significant difference between angry and happy expressions [*t*_(83)_ = 3.41, *p* = 0.001], neutral vs. happy differences were just short of significance [*t*_(83)_ = 1.98, *p* = 0.05]. However, neutral and angry expressions did not differ [*t*_(83)_ = 1.44, *p* = 0.15].

#### Error rates

There was a main effect of condition [*F*_(1, 83)_ = 150.45, *p* < 0.001], reflecting higher errors on antisaccade trials (*M* = 18%, *SD* = 11) compared to prosaccade (*M* = 5%, *SD* = 6). There was also a main effect of load [*F*_(1, 83)_ = 8.14, *p* < 0.01] indicating modest but significantly higher errors under high load (*M* = 12%, *SD* = 8) compared with low load (*M* = 11%, *SD* = 9). Furthermore, a significant two-way interaction of load × condition [*F*_(1, 83)_ = 8.56, *p* < 0.01] mirrored latency data by showing increased errors under high compared to low load on antisaccade trials [*M* = 16–20%; *t*_(83)_ = 3.40, *p* = 0.001], but no effect on prosaccade trials (*M* = 5–5%; *t* < 1).

There was no main effect of expression or interaction with load (*F*'s < 1), or condition [*F*_(2, 166)_ = 1.13, *p* = 0.33]. However, a Three-Way interaction of expression × load × condition was observed [*F*_(2, 166)_ = 5.77, *p* < 0.01]. Separate ANOVAs within each level of condition factor showed a trend under prosaccade trials for a main effect of expression [*F*_(2, 166)_ = 2.42, *p* = 0.09], and for an interaction of expression × load [*F*_(2, 166)_ = 2.71, *p* = 0.07]. On antisaccade trials, there was no main effect of expression (*F* < 1) but a significant expression × load interaction [*F*_(2, 166)_ = 3.17, *p* < 0.05]. While there was no effect of expression under high load (*F* < 1), an effect in low load was seen [*F*_(2, 166)_ = 3.74, *p* < 0.03]. Similar to response latencies, Table 1 shows the lowest errors on happy expression trials, with the highest on angry expression trials, reflected by a strong linear contrast [*F*_(1, 83)_ = 9.09, *p* < 0.01]. Pairwise comparisons showed that errors on angry and happy expression trials significantly differed [*t*_(83)_ = 3.02, *p* < 0.01]. Differences between neutral and angry expression trials did not reach significance [*t*_(83)_ = 1.70, *p* = 0.09], and nor did neutral vs. happy expression trial error rates [*t*_(83)_ = 1.02, *p* = 0.31].

### Effects of individual differences in trait anxiety

#### Saccade latencies

**Figure 2:**
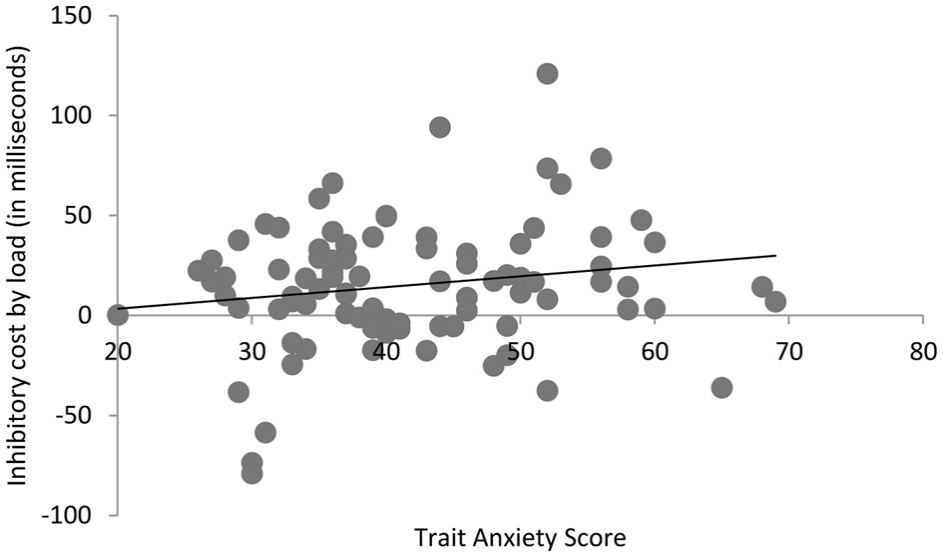
**Correlation between trait anxiety score and load-induced inhibitory costs on saccadic eye-movements**.

Trait anxiety scores varied across participants (Med = 39, *SD* = 11, range = 20–69). As occulomotor speed differs between individuals, we subtracted prosaccade latencies from antisaccade for each participant, creating a measure of cost to saccade speed on trials requiring a controlled eye-movement vs. a baseline reflexive response. Using trait anxiety score as a continuous measure, we then correlated this with inhibitory cost from the low and high load conditions. Under low load, there was no significant correlation between trait anxiety score and inhibitory costs (*r* = 0.105, *N* = 84, *p* = 0.34). However, anxiety was associated with inhibitory costs under high load (*r* = 0.244, *N* = 84, *p* < 0.03). Furthermore, when subtracting the inhibitory cost under low load from high to reveal the extent of load in disrupting inhibition, a positive correlation with anxiety again emerged (*r* = 0.182, *N* = 84, *p* < 0.05 one-tailed; see Figure 2). Thus, anxiety was associated with the magnitude of cost on inhibition under cognitive load.

To assess whether the effect of anxiety and inhibition was modulated by the emotional expression of the visual onset, we conducted further correlational analyses with anxiety and inhibitory costs for each expression under each level of load. Under low load, anxiety did not correlate with inhibitory costs on neutral (*r* = 0.150, *N* = 84, *p* = 0.17) or either emotional expression level (*r*'s < 1). Under high load, evidence of a positive correlation between inhibitory costs and anxiety was observed regardless of expression being neutral (*r* = 0.269, *N* = 84, *p* = 0.01), angry (*r* = 0.186, *N* = 84, *p* = 0.05 one-tailed), or happy (*r* = 0.179, *N* = 84, *p* = 0.05 one-tailed). Modulations by anxiety therefore appeared to occur regardless of stimulus emotion.

#### Error rates

Anxiety score did not correlate with inhibitory cost error rates under either level of load (*r*'s < 1), and did not correlate with the load cost (*r* = −0.107, *N* = 84, *p* = 0.33). Likewise, there was no evidence of anxiety associated with inhibitory costs for different emotional expressions at any load level (all *r*'s < 0.148, *p*'s > 0.18).

## Discussion

Results from the present study suggest that increasing cognitive load not only disrupts attentional control processes, but this effect is especially potent for individuals with high levels of trait anxiety. Furthermore, results show an effect of emotional valence by load, in that evidence of emotion modulating task performance was evident on antisaccade trials only under low load; high load eliminated all emotional expression differences.

### Attentional control in trait anxiety under cognitive load

Deficits in attentional control in trait anxiety, particularly on the subcomponent of cognitive inhibition, have been suggested in a number of previous investigations examining covert (e.g., Pacheco-Unguetti et al., 2010) and overt (e.g., Derakshan et al., 2009) attention, with additional electrophysiological and neuroimaging studies suggesting individual differences in the prefrontal attentional control network (Bishop, 2009; Ansari and Derakshan, 2011a). However, ACT's prediction that the cognitive demands in a task should hamper performance to a greater extent in individuals with pre-existing deficits in attentional control has to date received far less empirical support. Previous evidence for this theory has primarily come from language-based experiments, where anxious individuals perform worse under more difficult test conditions (Calvo, 1985; Calvo et al., 1992) or when cognitive load is manipulated through digit rehearsal of increasing difficulty (MacLeod and Donnellan, 1993; Derakshan and Eysenck, 1998; Wood et al., 2001) during tasks that assess grammatical reasoning. However, as with a recent study by Berggren et al. (2012), described in our Introduction, these studies cannot clearly show that attentional control in anxiety was further disrupted by load, as increased costs could be due to general issues in performance and not attributable to attentional control *per se*. The present study rules out this possibility; anxious individuals did not perform worse on prosaccade trials under load, where no element of inhibition is required to be efficient in the task. On the other hand, performance on antisaccade trials requiring inhibitory control to suppress a reflexive eye-movement was impaired under load, and the extent of this impairment directly correlated with participants' anxiety levels.

While the present study argues that anxious individuals should exercise poorer attentional control as a task's difficulty increases, it is worthy to address claims that the opposite may be the case. For example, Bishop (2009) showed that increasing perceptual task demands eliminated task-irrelevant distraction and individual differences in anxiety modulated distraction under low perceptual load. While this effect can be attributed to perceptual load reducing basic perception of distractors (see Berggren and Derakshan, 2013, for comment), it was noted that attentional focus could play a role in the effect of anxiety on attentional control. In other words, under low attentional demands, anxious individuals may be more distracted but this effect wanes as more demanding tasks prompt focused attention. Similarly, it has been suggested that working memory capacity/span relates to attentional control abilities. While there have been many demonstrations of attentional control being disrupted by taxing working memory, it has also been documented that individuals with low working memory span are less susceptible to cognitive load manipulations (Kane and Engle, 2000).

One explanation for this finding is that anxious individuals already have impaired attentional control abilities, and using a load to tax inhibitory ability would have a reduced effect when baseline ability is already nearer floor. Importantly, in the present study, inhibitory ability between groups did not differ under low cognitive load; rather than group differences attenuating, they emerged as cognitive load was raised. Therefore, it is possible that attention focus in anxious individuals was enhanced in light of increased task demands but that high cognitive load taxed attentional control and counteracted this process. Whether or not factors such as motivation can impact attentional control deficits in low cognitively demanding tasks is a theoretically important question for future research. Indeed, according to one of the major predictions of ACT, motivation may play a key role in attentional control deficits in anxiety, with poor performance under low motivation and improved performance when encouraged (see Berggren and Derakshan, 2013).

### Emotion processing under cognitive load

Results from the present study also showed that cognitive load affected emotion processing. Under low load, emotional expression modulated both latencies and error rates, and this effect was confined to the antisaccade condition rather than prosaccade. As prosaccade performance is mainly reflexive, it is likely that the absence of an effect of emotional expression in this condition reflects latency speed being at ceiling. In the antisaccade condition, where participants should inhibit the reflexive eye-movement, there is greater scope for differences to emerge. Notably, such valence differences only occurred under low load; high load both slowed antisaccade latencies and eliminated differences between valence conditions. This finding is unlikely to be due to converse floor effects on latencies with the imposition of load, considering that individual differences in anxiety influenced performance under high load.

Our findings with emotion and cognitive load mirror that of a number of previous studies that have primarily examined interactions of load with negative emotions. Van Dillen and Koole (2009) found that angry, compared to happy, distractors slowed reaction times under low load, but did not differ under high load. Emotional startle effects from threatening images has also been shown to be reduced under cognitive load, as has LPP amplitude reflecting emotional arousal (e.g., MacNamara et al., 2011). Even neural activity in the amygdala, seen as a clear index of the processing of negative emotion, has been suggested to show weaker response to emotion under cognitive load or distraction techniques (Van Dillen et al., 2009; McRae et al., 2010). Collectively, these results support the view that the processing of emotional information may share resources with other cognitive processes (e.g., Pessoa, 2010).

However, the effects by emotion observed in the present study did not appear solely driven by the threat value of angry expressions. Differences between happy and angry expression trials were seen for both response latencies and error rates, but comparison with neutral expression trials acting as a baseline did not differ from the other emotional conditions significantly for latencies or error rates. Consequently, the present study supports the view that cognitive load can reduce the impact of emotional stimuli on cognitive processes such as inhibitory control, but cannot clarify whether low load effects were predominantly caused by negative emotion impairing performance or perhaps positive emotion facilitating it. While there is ample evidence that positive emotional stimuli are also prioritized for attention (e.g., Williams et al., 2005), we avoid speculation given that the locus of emotion modulations are unclear here; our hypothesis was concerned with whether cognitive load would attenuate emotional influences on inhibitory control, which was supported.

Finally, we did not observe any differential biases toward threat in anxiety under low cognitive load, despite a wealth of previous literature supporting such a bias for highly anxious groups (see Bar-Haim et al., 2007). A similar finding was also obtained in Berggren et al. (2012) where anxiety led to performance costs under cognitive load but did not adversely affect emotion processing in general. One probable explanation for this result is that threat biases overall were weak when making comparisons with neutral expression trials. In other words, effects by anxiety may not have been evident due to the threat value of angry stimuli not being sufficiently high enough in this experimental context to elicit biases for high anxious participants. Thus, it remains unanswered here how inhibitory control of irrelevant *threat* stimuli in anxiety is affected by loading cognitive processes.

### Future directions

While the present study establishes clear evidence that trait anxiety results in poor attentional control under high cognitive demands, a number of avenues for future research remain. Firstly, previous studies examining attention to threat in anxiety under cognitive load have found conflicting results, with some indication of enhanced threat processing (Ladouceur et al., 2009; Judah et al., 2013), smaller reductions in LPP arousal responses in anxiety (MacNamara et al., 2011), but also reduced emotional startle for high anxious participants under load (Dvorak-Bertsch et al., 2007; Vytal et al., 2012). It is noteworthy that these studies can be separated reasonably well in relation to their measure of anxiety; increased threat biases seem to occur when trait anxiety is examined (Ladouceur et al., 2009; Judah et al., 2013), while reduced emotional effects seem to dovetail studies where anxiety has been induced in participants (Dvorak-Bertsch et al., 2007; Vytal et al., 2012; but see MacNamara et al., 2011).

Both trait anxiety and mood induction of state anxiety, such as through threat of shock, have been shown to have similar effects on distractibility in some contexts such as the antisaccade task (Cornwell et al., 2012), but may do so through different means. For example, Pacheco-Unguetti et al. (2010) found that trait anxiety reduces executive control of attention while state anxiety modulates the alerting and orienting functions. Thus, the effect of cognitive load may differ in that it exacerbates behavioral effects for trait anxious participants while alleviating effects of state mood. Indeed, it is a possibility that cognitive load could attenuate the priming aspect of a mood induction, while having little effect on fundamental neural differences associated with a trait anxious personality. Future research should examine whether cognitive load can be a beneficial therapy intervention in reducing unwanted emotional experience, as proposed by some (e.g., Van Dillen et al., 2009), or whether it can conversely be detrimental to emotion regulation. The type of anxiety experienced, whether trait or state, could be a crucial factor in this regard. This is particularly important to clarify considering that trait anxiety is a major vulnerability factor in the development of pathological anxiety disorders.

Secondly, future research may further examine how cognitive load impairs more general attentional control in anxiety. Here, we have suggested that anxiety exacerbates cognitive load effects on attention, but it remains unclear how this influences across tasks. As we did not record accuracy for our secondary auditory task, it is possible that cognitive load may have impaired performance on both tasks, further compromising attentional control in anxiety. Furthermore, individual differences in response to emotional stimuli may have been evident on the secondary load task, with anxious participants prioritizing the saccade task in such instances and reducing accuracy and/or response times to the tones. Further work should examine how high anxious individuals coordinate their resources under dual task conditions, as well as the effect of divided attention paradigms. This would also provide more insight into the underlying neural mechanisms behind the present results; previous work has highlighted that anxiety modulates areas associated with attentional control such as dorsolateral prefrontal cortex (Bishop, 2009; Basten et al., 2011), and cognitive load has been shown to increase visual representations for distractor information (e.g., de Fockert et al., 2001). On this basis, the ability to suppress distractor representations under load may have been more strongly impaired in anxious individuals. How cognitive load effects may translate in anxiety under dual task conditions requiring more internal suppression of task goals when switching between tasks remains an open question.

## Conclusion

The present study suggests that increasing cognitive load disrupts performance in tasks requiring attentional control, particularly for individuals reporting high levels of trait anxiety. This supports ACT's prediction that increasing task demand causes greater attentional control decrements in high anxiety, pointing to a poorer ability to maintain task goals when pre-existing deficits in attentional control are further compromised. Finally, results also suggest reduced threat biases in attention under cognitive load, supporting accounts of shared emotion-cognition resources.

### Conflict of interest statement

The authors declare that the research was conducted in the absence of any commercial or financial relationships that could be construed as a potential conflict of interest.

## Footnotes

^1^The STAI has high internal consistency in measuring trait anxiety. In the present study, the spread of anxiety scores is consistent with previous work, where medians tend to sit around a score of 37 (Fox et al., 2002). Furthermore, as illustrated in Figure 2, we had an adequate proportion of scorers below 35 and above 40, suggested to be appropriate cut-offs to reflect clear low and highly anxious individuals (Fox et al., 2002).
